# Characterization of Rett Syndrome-like phenotypes in *Mecp2*-knockout rats

**DOI:** 10.1186/s11689-016-9156-7

**Published:** 2016-06-16

**Authors:** Yang Wu, Weiwei Zhong, Ningren Cui, Christopher M. Johnson, Hao Xing, Shuang Zhang, Chun Jiang

**Affiliations:** Department of Biology, Georgia State University, 50 Decatur Street, Atlanta, GA 30302 USA

**Keywords:** *Mecp2*-null rat, Rett syndrome, Behaviors, Breathing, Locus coeruleus

## Abstract

**Background:**

Rett Syndrome (RTT) is a neurodevelopmental disease caused by the disruption of the *MECP2* gene. Several mouse models of RTT have been developed with *Mecp2* disruptions. Although the mouse models are widely used in RTT research, results obtained need to be validated in other species. Therefore, we performed these studies to characterize phenotypes of a novel *Mecp2*^−/Y^ rat model and compared them with the *Mecp2*^*tm1.1Bird*^ mouse model of RTT.

**Methods:**

RTT-like phenotypes were systematically studied and compared between *Mecp2*^−/Y^ rats and *Mecp2*^−/Y^ mice. In-cage conditions of the rats were monitored. Grip strength and spontaneous locomotion were used to evaluate the motor function. Three-chamber test was performed to show autism-type behaviors. Breathing activity was recorded with the plethysmograph. Individual neurons in the locus coeruleus (LC) were studied in the whole-cell current clamp. The lifespan of the rats was determined with their survival time.

**Results:**

*Mecp2*^−/Y^ rats displayed growth retardation, malocclusion, and lack of movements, while hindlimb clasping was not seen. They had weaker forelimb grip strength and a lower rate of locomotion than the WT littermates. Defects in social interaction with other rats were obvious. Breathing frequency variation and apnea in the null rats were significantly higher than in the WT. LC neurons in the null rats showed excessive firing activity. A half of the null rats died in 2 months. Most of the RTT-like symptoms were comparable to those seen in *Mecp2*^−/Y^ mice, while some appeared more or less severe. The findings that most RTT-like symptoms exist in the rat model with moderate variations and differences from the mouse models support the usefulness of both *Mecp2*^−/Y^ rodent models.

**Conclusions:**

The novel *Mecp2*^−/Y^ rat model recapitulated numerous RTT-like symptoms as *Mecp2*^−/Y^ mouse models did, which makes it a valuable alternative model in the RTT studies when the body size matters.

## Background

Rett syndrome (RTT), a progressive neurodevelopmental disorder, affects predominantly female children worldwide with an incidence of 1: 10,000 female births. People with RTT display a developmental regression which is characterized by a loss of acquired functions such as speech and hand skills, and development of stereotypic hand movements, gait apraxia, ataxia, and seizures [1–3]. Breathing disorders are common including hyperventilation, apnea, hyperpnoea, breath-holding, etc. [4–7], which contribute to the high incidence of sudden death.

Defects in the X-linked gene *MECP2* encoding the methyl CpG binding protein 2 (MeCP2) known as a transcriptional regulator, are identified in more than 90 % RTT patients. Mutations or altered expression of *Mecp2* in mice result in a spectrum of RTT-like neurodevelopmental disorders. Thus, several mouse models of RTT have been developed and used in RTT studies over the past years [8, 9]. For example, *Mecp2*^*tm1.1Bird*^, *Mecp*^*2Jae*^, and *Mecp2*^*neoTam*^ mouse models with exon 3 and/or 4 deletions in the *Mecp2* are widely used in the etiologic and therapeutic studies [9–11]. Other mouse models with site-specific mutations, such as *Mecp2*^*T158A/y*^, *Mecp2*^*R168X*^, and *Mecp2e1*^−/Y^, were developed in recent years [12–14]. By using these mouse models, considerable progress has been made in the studies of the neurobiological mechanisms underlying or associated with RTT [15–19]. *Mecp2*^*tm1.1Bird*^ mice, the most widely used mouse model of RTT, show several phenotypes similar to human patients with RTT, such as early death, motor problems, and autistic behaviors, which progressively worsen until death [20, 21]. Autonomic dysfunction is another characteristic manifestation including breathing disorders, cardiac arrhythmias, and constipation [5, 22, 23]. Brainstem monoamine modulatory systems are involved. Electrophysiological and molecular biological studies in the *Mecp2*^*tm1.1Bird*^ mouse model suggest that neurons in the locus coeruleus (LC) are defective, showing impaired neuronal activity, abnormal CO_2_ chemosensitivity and reduced expression of enzymes for NE biosynthesis [19, 23–25].

Although the mouse models are widely used in RTT research, results obtained need to be validated in other species, which is particularly important when therapeutic modalities are aimed to develop. Thus, studies on multiple animal models of RTT are necessary and highly significant. A novel *Mecp2*-knockout rat model, SD-*Mecp2*^tm1sage^ rat, has been developed recently (see below). The null rats had a deletion of exon 4 in the *Mecp2* leading to complete elimination of the MeCP2 protein. Since the rat model may provide useful information on RTT mechanisms and treatments, we performed systematic studies of the RTT-like symptoms identified previously in *Mecp2*^−/Y^ mouse models, and compared these RTT-like phenotypes with those found in the male mouse model of RTT.

## Methods

### Animal

All animal experiments were conducted in accordance with the National Institutes of Health (NIH) *Guide for the Care and Use of Laboratory Animals* and were approved by the Georgia State University Institutional Animal Care and Use Committee. The experiments were performed on male *Mecp2*^−/Y^ rats because the male model offers a completely *Mecp2*-null condition that is not always available in the female owing to random X-chromosome inactivation [26, 27]. To breed these males, the female heterozygous *Mecp2*^*+*/−^ rat (SD-*Mecp2*^*tm1sage*^, strain code: TGRA6090) from the supplier company Horizon Discovery Group (Boyertown, PA) were crossbred with the male Sprague-Dawley wild-type rat (WT). The genotyping protocol from Sage Labs in Horizon Discovery Group was used to identify WT, heterozygous, and homozygous animals based on a 71 bp deletion in the *Mecp2* (Primer F: GCAGCATCAGAAGGTGTTCA, Primer R: GACCTCAATGCTGACGGTTT). Female heterozygous mice (Genotype: *Mecp2*^*+*/−^; Strain name: B6.129P2(C)-*Mecp2*^tm1.1Bird^/J; Stock number 003890) from Jackson Lab were crossbred with male C57BL/6 mice to produce the *Mecp2*^−/Y^.

### Body weight and in-cage conditions

Body weight of animals was measured and recorded every week starting from postnatal day 7 (P1w) till death. Teeth growth, food and water intake, movements, and other body conditions were regularly checked as well. Overly grown incisors that affected the food intake were monitored and trimmed by the veterinarian weekly.

### Grip strength

During the experiment, an animal was lifted by the tail, and their forelimbs were allowed to grasp the metal mesh fixed to a force-electricity transducer (CB Sciences, Inc., Milford, MA). The animal was then gently pulled upward while it grasped the mesh (10 mm × 10 mm) with both forelimbs. The maximal force reached immediately before it released the mesh was taken as the grip strength. Signals were amplified, collected with the Clampex 9 software, and stored in a computer. Comparison between rats and mice was done in animals aged 5–6 weeks.

### Spontaneous locomotion test

Locomotion activity of male rats aged 4–5 weeks was tested in a test chamber made of white plexiglass boards (80 cm × 80 cm × 30 cm). The arenas were divided into 10 cm × 10 cm squares (*n* = 64) by drawing thin black lines on the floor. The locomotion apparatus for male mice aged 5–6 weeks was constructed with the same material in size 50 cm × 50 cm × 30 cm with 10 cm × 10 cm square lines. Animals were carried to the test room in their home cages and habituated for 30 min before the locomotion test. Each tested animal was placed in a center of the box and observed for 5 min. Each trail was recorded with a video camera. Measures of square crosses (all four paws cross) were obtained from the video record. After the test, the apparatus was thoroughly cleaned using 70 % ethanol and then H_2_O to eliminate potential residual odors and contaminants.

### Three-chamber test

WT and *Mecp2*^−/Y^ male rats, aged 4-5 weeks, were tested in a three-chambered apparatus (90 cm × 60 cm × 30 cm), where three chambers (30 cm × 60 cm × 30 cm each) were separated by two transparent plexiglass walls with an opening in each wall. Two buckets (12 cm in diameter × 20 cm in height) made of 1 cm × 1 cm metal mesh were placed in the center of the two-side chambers. Similarly, mice aged 6–7 weeks were tested in an apparatus with three 20 cm × 30 cm × 40 cm chambers. The buckets and the metal mesh were 1/3 smaller in mice than in rats. On the test day, animals were allowed for 30 min habituation to its environment. A three-step procedure was performed. First, the tested animal was placed in the center chamber, and allowed to freely move over all three chambers for 10 min. The time spent in each chamber was recorded with a video camera. Second, sociability was tested, in which a male intruder (Animal 1) in one mesh bucket was introduced to one of the side chambers randomly, while the other bucket was kept empty. The test animal was allowed to explore both the chambers for 10 min with the total time spent in the chamber measured. The intruder was randomly assigned in one of the side chambers to avoid the side bias. Lastly, the social novelty was tested by switching the familiar intruder into the other chamber and introducing a novel male intruder (Animal 2) to the chamber. The tested animal was monitored for 10 min as well. The time spent in each side chamber was measured. The apparatus was thoroughly cleaned between trails using 70 % ethanol and H_2_O. All behavior tests were performed during the light cycle (10 am to 7 pm).

### Plethysmograph Recording

Breathing activities of awake rats were recorded with a ~1100-ml plethysmograph chamber. The individual animal was kept in the plethysmograph chamber with air ventilation at a rate of ~1000 ml/min. After at least 20 min adaptation, breathing activity was recorded continuously for 20 min as the barometrical changes between the plethysmograph chamber and the reference chamber with a force-electricity transducer (Emka, Oxford, UK). The signal was amplified and recorded with AxoScope 10.3 software in a computer. The data was analyzed with Clampfit 10.3 software. Apnea rate and respiratory frequency variation were measured. Apnea was considered only if the inspiration interval was twice longer than the previous breathing. Breathing frequency variation was defined as the standard deviation (SD) of the breathing frequency divided by their arithmetic mean. The SD and arithmetic mean were measured from ~200 successive breathing events.

### Electrophysiology

The electrophysiological experiments were done as we described previously [24]. In brief, experimental rats or mice were decapitated after deep anesthesia with inhalation of saturated isoflurane. The brainstem was obtained and immediately placed in an ice-cold and sucrose-rich cutting (in mM) solution containing 220 sucrose, 33 NaHCO_3_, 1.9 KCl, 6 MgCl_2_, 1.2 NaH_2_PO_4_, 0.5 CaCl_2_, and 10 d-glucose. The solution was bubbled with 95 % O_2_—5 % CO_2_ (pH 7. 40). Transverse pontine sections (150–300 μm) containing the LC were obtained by using a vibratome sectioning system and then recovered at 33 °C for 60 min in normal artificial cerebrospinal fluid (aCSF) (in mM) solution containing 124 NaCl, 26 NaHCO_3_, 3 KCl, 2 MgCl_2_, 1.3 NaH_2_PO_4_, 2 CaCl_2_, and 10 d-glucose. The brain slices were kept at room temperature before usage. At recording, the slices were perfused with oxygenated aCSF at a rate of 2 ml/min and maintained at 34 °C in a recording chamber by a dual automatic temperature control (Warner Instruments).

Whole-cell current clamp was performed on LC neurons in the brain slices. Patch pipettes with a resistance of 3–5 MΩ were prepared with Sutter pipette puller (Model P-97, Novato, CA). Only were the neurons with membrane potential less than −40 mV and action potential larger than 65 mV accepted for further experiments. The pipette solution (in mM) contained 130 K gluconate, 10 KCl, 10 HEPES, 2 Mg-ATP, 0.4 EGTA, and 0.3 Na-GTP (pH 7. 3). The bath solution was normal aCSF bubbled with 95 % O_2_ balanced with 5 % CO_2_ (pH 7. 40). Recorded signals were amplified with an amplifier Axopatch 200B (Molecular Devices, Union City, CA), digitized at 10 kHz, filtered at 2 kHz, and collected with the Clampex 8.2 data acquisition software (Molecular Devices).

### Lifespan

WT and *Mecp2*^−/Y^ animals were randomly selected from litters. Their lifespan were monitored. Death date of each group was recorded. Both in-cage death and the cases reach humane endpoint determined by the animal facility at Georgia State University were counted. One outlier at each end was removed from the data analysis to minimize the data variation.

### Randomization/double blind

The intruders used in the three-chamber test were randomly selected from same species WT animals, and randomly placed into one of the side chambers in the sociability session. All the behavior experiments, including motor function, social behavior, and breathing activity were done double-blindly by two to three people. All patch experiments were done double-blindly by three people.

### Data analysis

Data are presented as means ± SE. Student’s *t* test was used to perform the statistical analysis. Difference was considered significant when *P* ≤ 0.05 with degree of freedom (df) shown.

## Results

### General RTT features

**Fig. 1:**
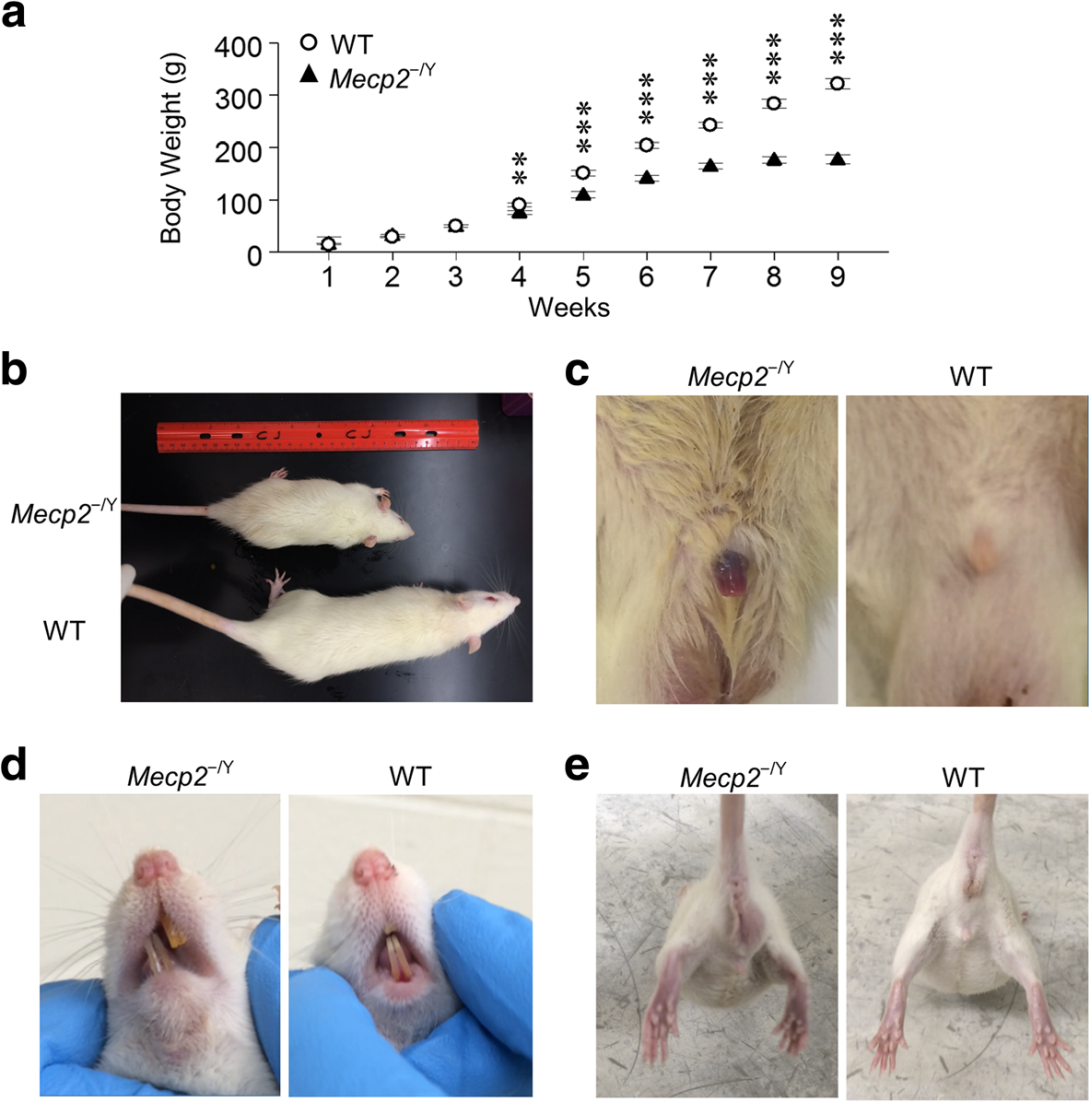
General abnormalities of *Mecp2* ^−/Y^ rats. **a** Body weight of *Mecp2* ^−/Y^ rats was significantly lower than that of WT littermates at P4w, and the difference became larger with growth (***P < 0.001, **P < 0.01; Student’s *t* test). *Mecp2* ^−/Y^ rats also showed growth retardation (**b**), prolapsed penis (**c**), and malocclusion (**d**). **e** Typical hindlimb grasping seen in *Mecp2* ^−/Y^ mice was not observed in *Mecp2* ^−/Y^ rats

Mutation of the *Mecp2* gene in rats caused several developmental abnormalities. Some of the abnormalities have previously been identified in *Mecp2*^−/Y^ mice, and others seemed more or less obvious in rats than in mice. The body weight of *Mecp2*^−/Y^ rats was monitored every week starting from postnatal day 7 (P7) till death. In comparison to their WT littermates, *Mecp2*^−/Y^ rats had a much smaller body size and lower body weight. When the body weight was compared between *Mecp2*^−/Y^ and WT rats, significant difference was observed in week 4 (75.6 ± 16.6 g in 18 *Mecp2*^−/Y^ rats vs 90.3 ± 14.8 g in 20 WT; *P* = 0.004, df = 41, *t* = −3.057), and the difference increased with growth (Fig. 1a, b). In addition, adult *Mecp2*^−/Y^ rats showed prolapsed penis (Fig. 1c) and severe malocclusion (overgrowth of incisor teeth and misplaced midline, Fig. 1d). Hindlimb grasping, a typical phonotype of *Mecp2*^−/Y^ mice, was absent in *Mecp2*^−/Y^ rats (Fig. 1e). The *Mecp2*^−/Y^ rats were also occasionally observed with porphyrin (red tear), which was not seen the WT littermates, and usually indicated sickness and stress.

### Muscle strength and locomotion

**Fig. 2:**
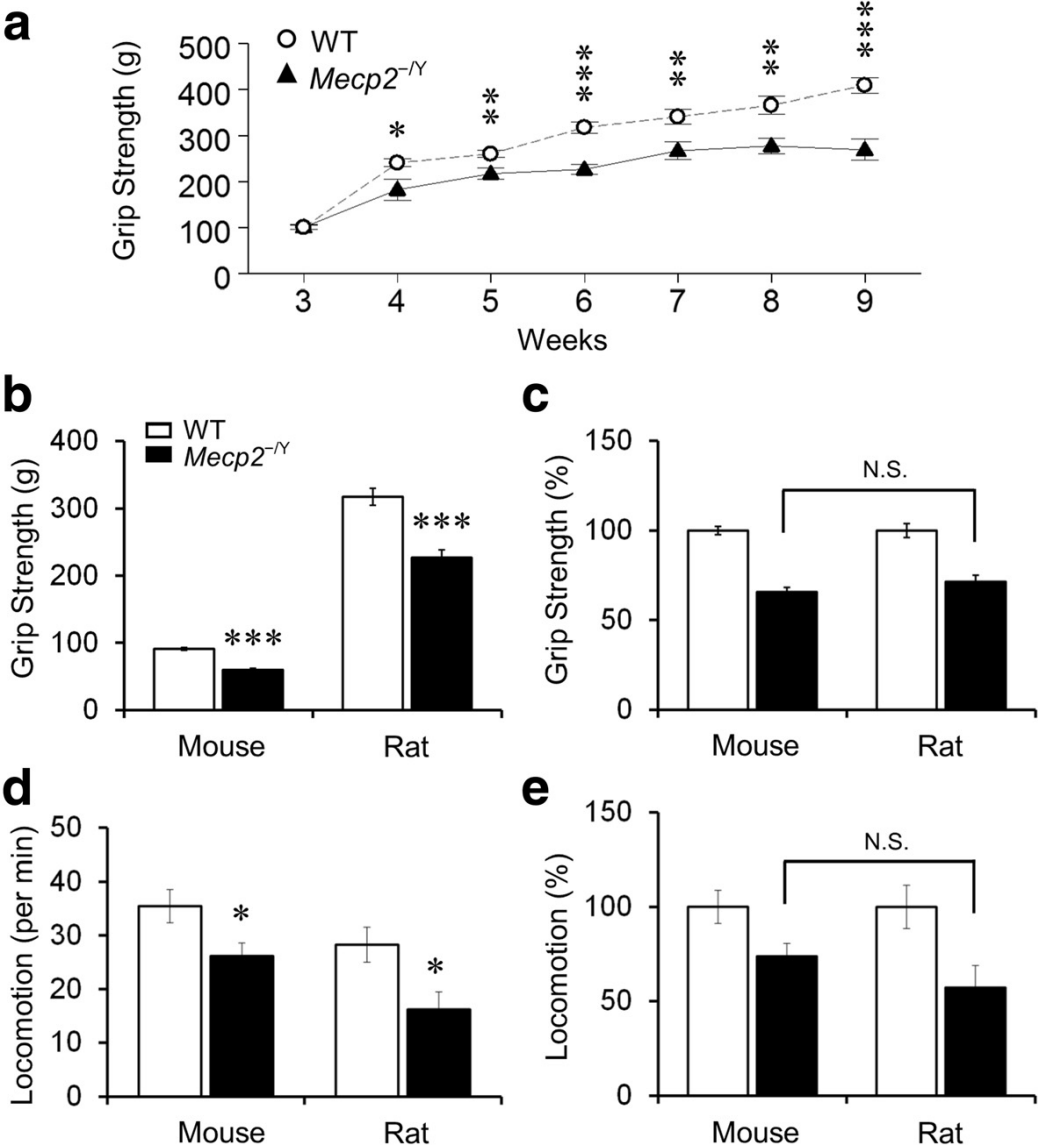
Reduced muscle strength and locomotor activity. **a** *Mecp2* ^−/Y^ rats exhibited decreased forelimb grip strength compared to WT (***P < 0.001, **P < 0.01, *P < 0.05; Student’s *t* test). **b** In comparison to the WT, both *Mecp2* ^−/Y^ rats and mice showed a significant decrease in grip strength (Student’s *t* test). **c** No statistical significance in the grip strength differences was found between species after normalization to the WT levels. **d**, **e** Locomotor activity was also significantly reduced in *Mecp2* ^−/Y^ rodents. No statistical significance was found between species either, although the null rats appeared to move less (Student’s *t* test)

People with RTT have defects in motor functions, some of which have been found in *Mecp2*^−/Y^ mice. Similarly, *Mecp2*^−/Y^ rats showed fewer movements than their WT littermates. Hence, we examined the motor function with grip strength test and the observation of spontaneous locomotion. Grip strength of the forelimbs was monitored weekly in 14 *Mecp2*^−/Y^ rats and 19 WT rats. The *Mecp2*^−/Y^ rats showed a significant decrease in muscle strength starting in 4 weeks after birth (P4w), and remained significantly lower thereafter than that of the WT (Fig. 2a). The maximum grip strength was reached in P7w in null rats. In comparison, the grip strength of WT rats continued to increase with growth.

When the grip strength was compared in P6w, the muscle strength of both *Mecp2*^−/Y^ rats and mice was significantly lower than that of their WT littermates (Fig. 2b). Because of the body size, rats were much stronger than mice. We thus normalized the grip strength to the WT in each group. After normalization, similar reductions in the muscle strength were observed in both *Mecp2*^−/Y^ rats (by 29 % of the WT value) and *Mecp2*^−/Y^ mice (by 34 %) with no statistical difference found between species (Fig. 2c).

The spontaneous movement was measured with traveling distance to indicate locomotor activity. A rat was placed in the center of an enclosure and allowed to explore around for 10 min. The number of the squares crossed by the rat was counted and recorded as locomotion activity (squares crossed per min). The less square traveling, the lower locomotor activity. The locomotion activity of both *Mecp2*^−/Y^ strains were significantly lower than the WT littermates (35.4 ± 3.1 squares/min in 9 WT mice vs 26.2 ± 2.4 squares/min in 9 *Mecp2*^−/Y^ mice, *P* = 0.031, df = 17, *t* = 2.367; 28.3 ± 3.3 squares/min in 12 WT rats vs 16.2 ± 3.3 squares/min in 11 *Mecp2*^−/Y^ rats; *P* = 0.016, df = 21, *t* = 2.609) (Fig. 2d). After normalization to the WT level, *Mecp2*^−/Y^ rats showed 43 % reduction in locomotor activity, while *Mecp2*^−/Y^ mice had 26 % reduction (Fig. 2e). The low locomotion activity of *Mecp2*^−/Y^ rats suggests that the *Mecp2* disruption causes motor dysfunction in both rats and mice with similar severity.

### Autism-like behavior

**Fig. 3:**
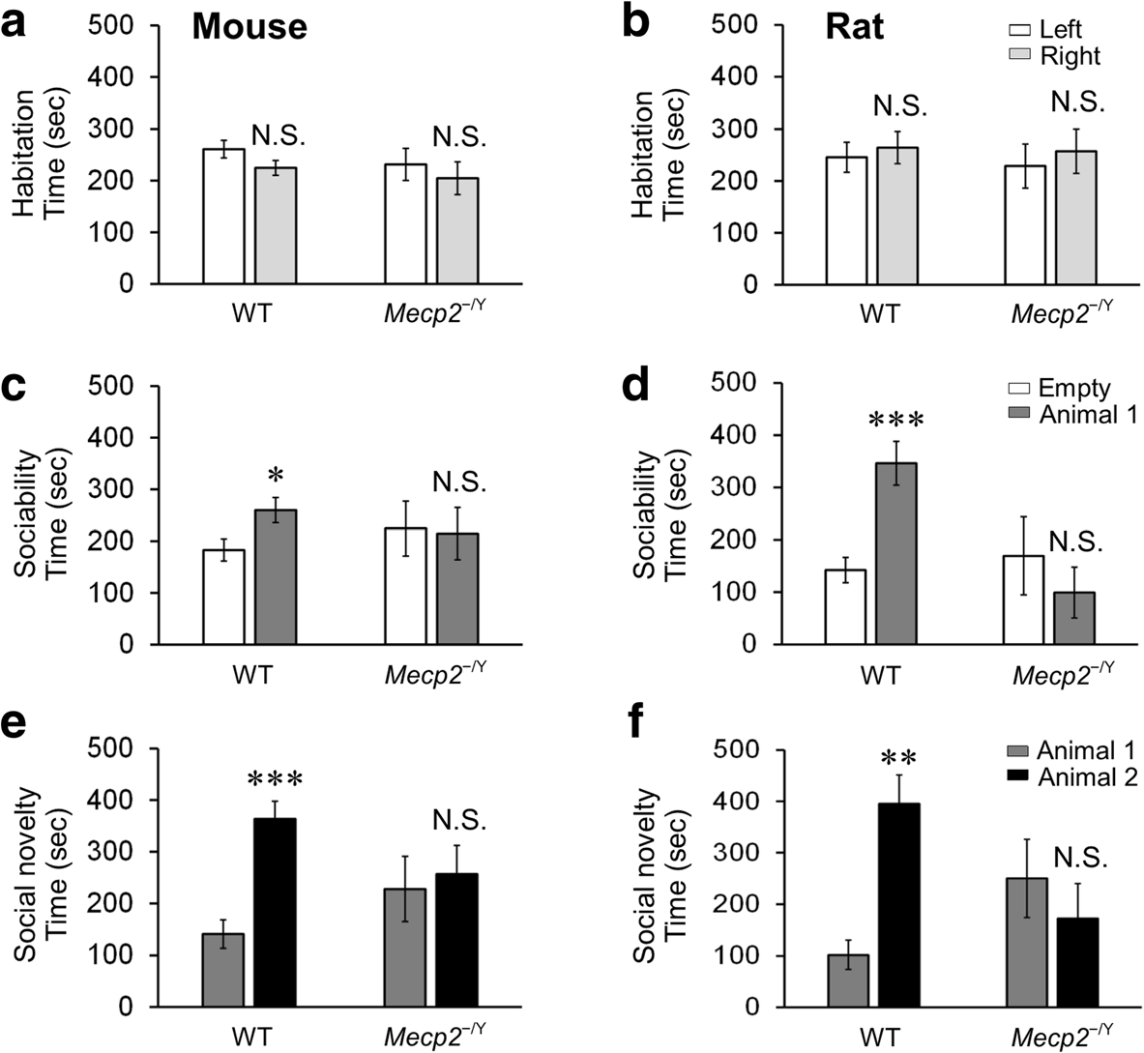
Social behavior defects of *Mecp2* ^−/Y^ rats. **a**, **b** The habituation time in side chambers had no significant difference in *Mecp2* ^−/Y^ animals and WT littermates. **c**, **d** The time spending of both *Mecp2* ^−/Y^ animals in the intruder chamber (*Animal 1*) was more than in the empty bucket chamber (*Empty*). **e**, **f** The animal preference disappeared in both *Mecp2* ^−/Y^ animals. Familiar animal is indicated as *Animal 1*, and unfamiliar animal is labeled as *Animal 2*. (***P < 0.001, **P < 0.01, * *P* < 0.05; Student’s *t* test)

Another clinic manifestation of RTT is the autism-like behavior that also occurs in *Mecp2*^−/Y^ mice. Using the three-chamber test, therefore, we studied the autism-like behavior in *Mecp2*^−/Y^ rats. During the tests, a rat was placed in the middle chamber of the three-compartmental box with an opening to each of the side chambers. During the first 10-min habituation period, rat preference to either side chamber was recorded as the time spent in each chamber (Fig. 3a, b). Only the animals showing no preference to either side of chambers in the habituation time were used for further tests.

Then, sociability was tested in a three-chamber apparatus with two identical mental-mesh buckets introduced to each side chamber. An intruder animal was randomly placed in one of the buckets, and the other was kept empty. The mesh bucket allowed the animal to have visual, olfactory, and auditory contact and exploration. During these second 10-min tests, WT rodents spent more time with their kinds than the empty chamber (259.8 ± 24.5 s vs 183.1 ± 21.3 s in 10 WT mice, *P* = 0.045, df = 19, *t* = 2.156; 346.4 ± 41.9 s vs 142.4 ± 24.2 s in 10 WT rats, *P* < 0.001, df = 19, *t* = -4.217) (Fig. 3c, d), consistent with previous reports [28–30]. Such a preference was not seen in both *Mecp2*^−/Y^ rodents. *Mecp2*^−/Y^ rats spent less time in the intruder chamber than in the empty one (99.2 ± 48.8 s with Animal 1 vs 169.5 ± 74.5 s in empty; *n* = 11, *P* = 0.439, df = 21, *t* = 0.789), similar to *Mecp2*^−/Y^ mice (214.5 ± 50.4 s with Animal 1 vs 224.4 ± 53.0 s in empty; *n* = 8, *P* = 0.895, df = 15, *t* = −0.135) (Fig. c, d). The results suggest that the defect of sociability was comparable between *Mecp2*^−/Y^ rats and mice.

The social novelty was tested during the last 10 min when a novel stranger (Animal 2) in addition to the first intruder (Animal 1 or familiar) was introduced to the previously empty bucket. The time spent in either side chambers was measured. Consistent with previous reports [29, 30], the WT animals showed a clear tendency of social novelty by spending a longer time with the stranger animal than with the familiar one (363.4 ± 34.5 s with Animal 2 vs 140.4 ± 27.5 s with Animal 1 vs in WT mice, *P* < 0.001, df = 19, *t* = 5.052; 395.4 ± 56.3 s with Animal 2 vs 101.8 ± 28.1 s with Animal 1 in WT rats, *P* = 0.002, df = 19, *t* = −4.666) (Fig. 3e, f). Such a preference disappeared in *Mecp2*^−/Y^ animals (227.7 ± 63.1 s with Animal 1 vs 256.7 ± 55.3 s with Animal 2 in *Mecp2*^−/Y^ mice, *P* = 0.422, df = 15, *t* = 0.828; 250.1 ± 76.0 s with Animal 1 vs 171.8 ± 68.1 s with Animal 2 in *Mecp2*^−/Y^ rats, *P* = 0.452, df = 21, *t* = 0.767) (Fig. 3e, f). Therefore, the results suggest that the *Mecp2*^−/Y^ rats show the autism-like social defects similarly to *Mecp2*^−/Y^ mice.

### Breathing abnormality

**Fig. 4:**
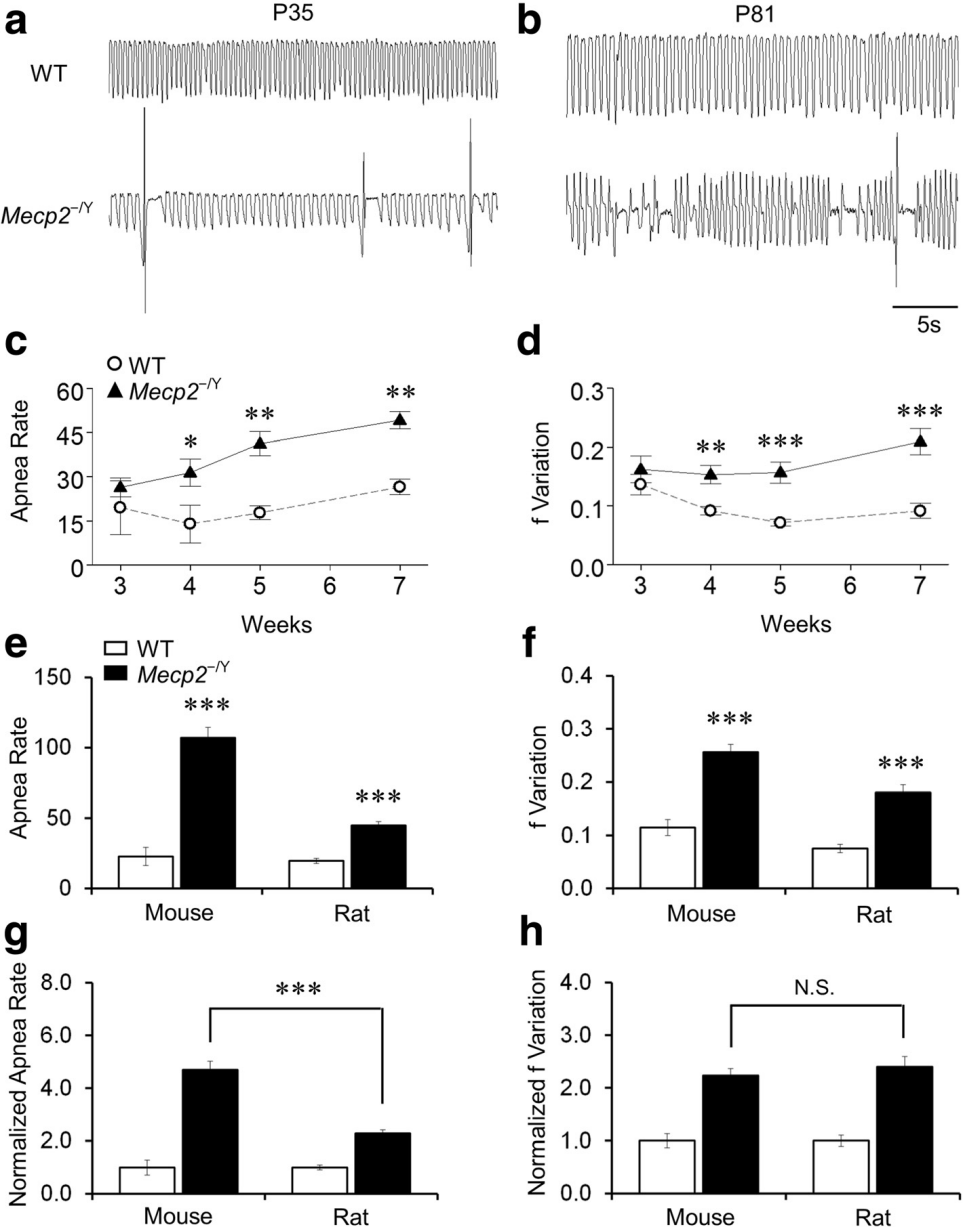
Breathing abnormalities. **a**, **b** Abnormal breathing activity including apnea and irregular breathing was observed in *Mecp2* ^−/Y^ rats at P35 and P81. **c**, **d** Apnea rate and respiratory frequency variation were significantly more in *Mecp2* ^−/Y^ rats than in the WT (***P < 0.001, **P < 0.01, *P < 0.05; Student’s *t* test). **e–h** the breathing abnormalities were compared between mouse and rat (***P < 0.001; Student’s *t* test). **g** The apnea rate of *Mecp2* ^−/Y^ rats was significantly lower than that of the *Mecp2* ^−/Y^ mice after data normalization to the WT levels. **h** The severity of breathing frequency variation was not significantly different between rats and mice

People with RTT as well as the mouse models of RTT show breathing abnormalities [4–7]. Thus, we measured the breathing activity using the plethysmography in conscious animals. Two most reliable breathing phenotypes, apneas and breathing frequency variations, were analyzed as shown in several previous studies [23, 31]. High apnea rate and breathing frequency variations were seen in *Mecp2*^−/Y^ rats. They started showing a significant difference from those in the WT rats in P4w, and deteriorated with growth (Fig. 4a–d). By P7w, the apnea rate was raised to 49.3 ± 2.9 h^−1^ of *Mecp2*^−/Y^ rats in comparison to 26.5 ± 2.7 h^−1^ of WT (Fig. 4c), and the breathing variation increased to 0.21 ± 0.02 in 15 *Mecp2*^−/Y^ rats comparing to 0.09 ± 0.01 in 8 WT (Fig. 4d). The breathing abnormalities of *Mecp2*^−/Y^ rats were compared with those in the *Mecp2*^−/Y^ mice at the same age group (5–7 weeks). Both *Mecp2*^−/Y^ strains had severe breathing problems in comparison to their WT littermates with a higher apnea rate (22.8 ± 6.5 h^–1^ in 12 WT mice vs 107.0 ± 7.4 h^–1^ in 24 *Mecp2*^−/Y^ mice, *P* < 0.001, df = 35, t = -7.335; 19.7 ± 1.8 h^–1^ in 11 WT rats vs 44.8 ± 2.7 h^–1^ in 27 *Mecp2*^−/Y^ rats, *P* < 0.001, df = 37, *t* = −5.784) (Fig. 4e) and a higher respiratory frequency variation (0.12 ± 0.02 in 14 WT mice vs 0.26 ± 0.02 in 25 *Mecp2*^−/Y^ mice, *P* < 0.001, df = 38, *t* = −6.184; 0.08 ± 0.01 in 11 WT rats vs 0.18 ± 0.02 in 27 *Mecp2*^−/Y^ rats, *P* < 0.001, df = 37, *t* = *−*4.454) (Fig. 4f). However, the apnea rate was significantly lower in *Mecp2*^−/Y^ rats than in the *Mecp2*^−/Y^ mice (*P* < 0.001, ANOVA and Student’s *t* test) (Fig. 4e, g), while the frequency variation had no significant difference between species (Fig. 4f, h). These results suggest that the breathing abnormalities seem less severe in *Mecp2*^−/Y^ rats than in *Mecp2*^−/Y^ mice.

### Hyperexcitability of LC neurons

**Fig. 5:**
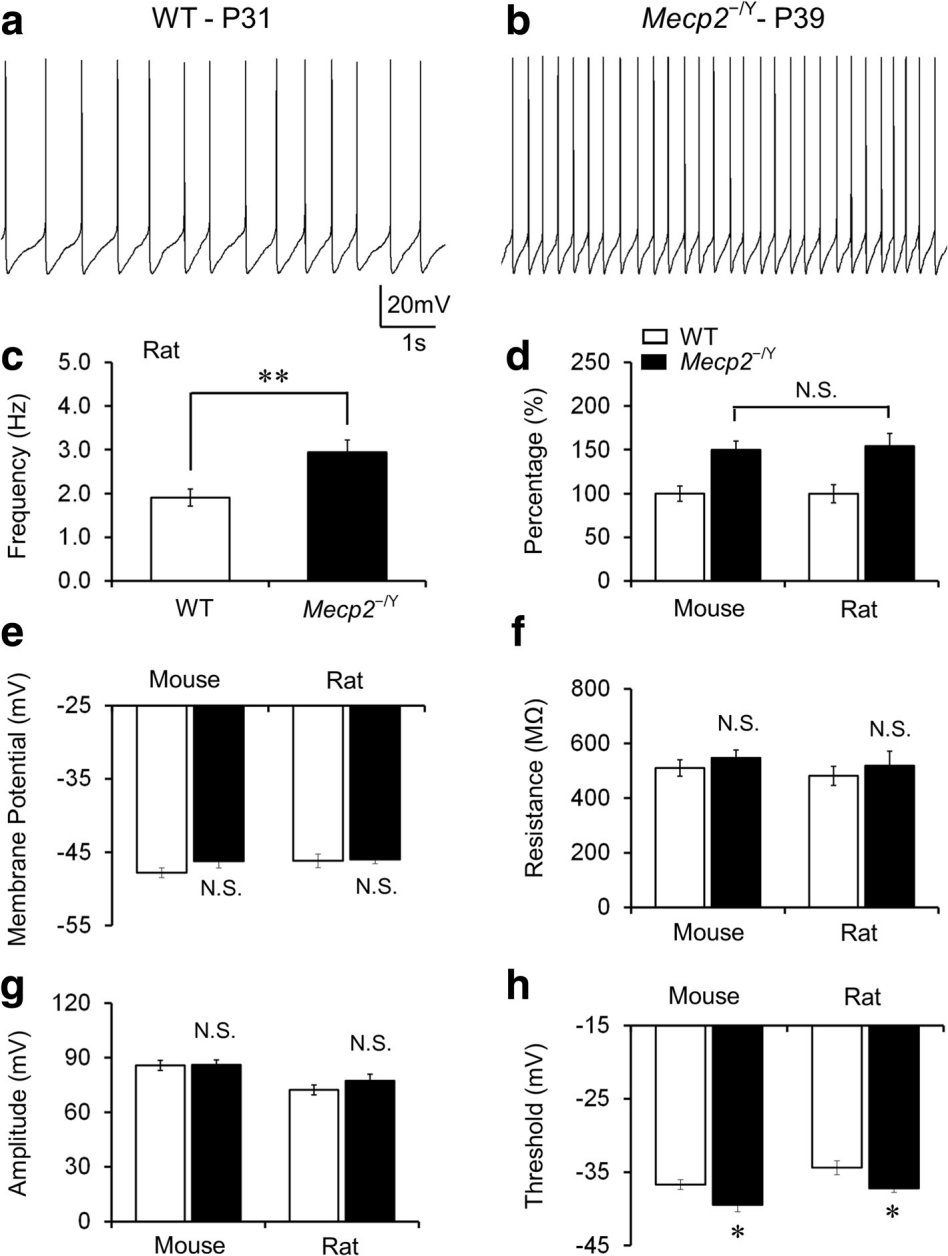
Increased excitability of LC neurons. **a, b** Typical recordings of spontaneous firing activity in WT and *Mecp2* ^−/Y^ rat LC neurons. **c** In *Mecp2* ^−/Y^ rats, LC neurons showed a significant increase in firing frequency in comparison to the WT. **d** The ratio of increased spontaneous firing rate, however, was similar between rats and mice. **e**, **f** No significant differences were found in membrane potential and resistance between WT and *Mecp2* ^−/Y^ rats, which was similar as in the mice model. **g**, **h** In comparison to the WT, *Mecp2* ^−/Y^ rats showed more hyperpolarized threshold of the action potential in LC neurons, whereas the amplitude was not altered. Similar results were found in the mouse model (**P < 0.01,*P < 0.05; Student’s *t* test)

Several groups of brainstem neurons are involved in breathing controls including those in the LC. Previous studies indicate that the *Mecp2* disruption in mice causes hyperexcitability in LC neurons [19, 32]. To test whether there is a change in LC neuronal excitability in *Mecp2*^−/Y^ rats, we studied the cells in brain slices. In whole-cell current clamp, LC neurons in *Mecp2*^−/Y^ rats (P4w-P6w) showed spontaneous firing rate 3.0 ± 0.3 Hz, which was significantly higher than the WT 1.9 ± 0.2 Hz (Fig. 5a–c. *P* = 0.006, df = 34, *t* = −2.921 Student’s *t* test). The firing rate of LC neurons in *Mecp2*^−/Y^ rats was increased by 54.0 ± 14.6 % over the WT level (Fig. 5c). Similar results were found in *Mecp2*^−/Y^ mouse model in the same age range as well (Fig. 5d). There were no differences in the membrane potential, input resistance, and action potential amplitude between *Mecp2*^−/Y^ and WT rats (Fig. 5e–g), neither between rats and mice. In *Mecp2*^−/Y^ rats, the threshold of action potential was shifted to significantly more hyperpolarizing potentials from −34.4 ± 1.2 to −37.2 ± 0.8 mV (Fig. 5h), which appeared to contribute to the increased firing activity of LC neurons.

### Lifespan

**Fig. 6:**
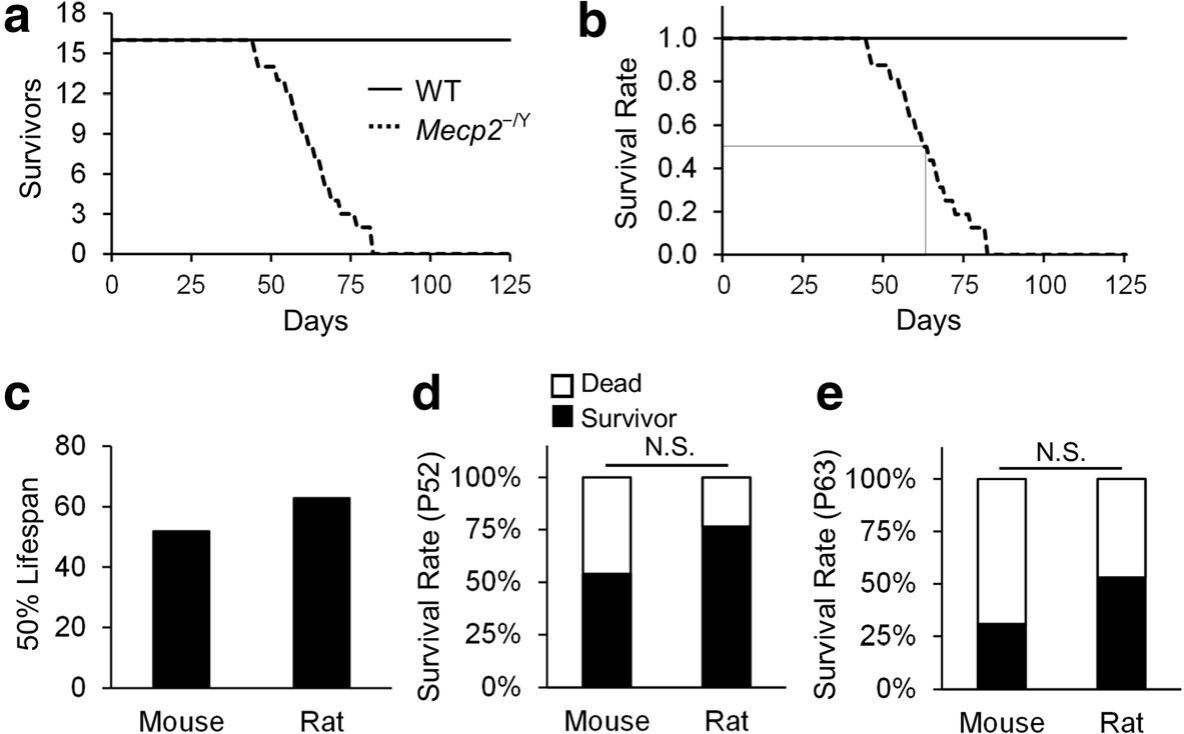
Survival rate. **a** The survival time of *Mecp2* ^−/Y^ rats was plotted in comparison to WT littermates (*n* = 16). **b** The 50 % lethality was shown after normalization of the initial rate to 1. **c** A half of *Mecp2* ^−/Y^ rats died at P63, while 50 % *Mecp2* ^−/Y^ mice dies at P52. **d** At P52, 50 % mice were survived, while about three fourth of the rats were still alive. **e** At P63 or the half survival age of *Mecp2* ^−/Y^ rats, only about one third of the mice were still alive. Despite these, no statistical significance was found between rats and mice (**d**, **e**)

These RTT-like symptoms may affect the lifespan of rats. Therefore, we examined the survival time in the novel *Mecp2*^−/Y^ rat model of RTT. The death was determined by either in-cage death or the humane endpoint determined by our school veterinarian. A total of 18 WT males and 18 *Mecp2*^−/Y^ rats were used in the studies, and two outliers (the longest and shortest survivors) were removed. Figure 6a shows that *Mecp2*^−/Y^ rats died as early as at P45, and all died by P82. Only two out of 16 tested rats survived beyond 80 days. Similar results were found in mice (not shown). In comparison, 50 % of the *Mecp2*^−/Y^ rats died at P63, while 50 % of the *Mecp2*^−/Y^ mice died at P52 (Fig. 6c). To compare these two groups of animals, we performed the *χ*^2^ test, and did not find any statistical difference in the ratio of survival vs death between these rats and mice at P52, neither at P63 (Fig. 6d, e).

## Discussion

RTT is a progressive neurodevelopmental disorder caused mostly by different mutations in the *MECP2* gene. The disease affects females mainly. Male infants rarely survive with pathogenic *MECP2* variations, because knockout of *MECP2* gene causes lethal miscarriages and early death in males [33, 34]. However, the male mice with *Mecp2* disruption survive, and show reliable and consistent RTT-like symptoms. This leads to wide uses of these mice in RTT studies. Experimental approaches to the female mouse models have limitations because of the X-chromosome inactivation and the large symptomatic variation among individuals. With the same consideration, our experiments were performed on the *Mecp2*^−/Y^ male rats as well. This novel RTT rat model has a 71-bp deletion in exon 4 at 53900-53970 of the *Mecp2* gene by Zinc Finger Nuclease technology. This results in a fragment deletion as well as a shift of the open reading frame leading to a complete knockout of MeCP2 [35]. Consistently, our studies demonstrate that the male *Mecp2*-null rats experience severe RTT-like symptoms.

### Recapitulation of RTT-like phenotypes in *Mecp2*^−/Y^ rats

Many general RTT-like features have been recapitulated in the rat model of RTT, including growth retardation, malocclusion, lack of movement, and early death. The *Mecp2*^−/Y^ rats show weaker forelimb grip strength and less locomotion activity than the WT littermates, which are similar to those observed in their mouse counterparts. Defects in sociability and social novelty were seen in these *Mecp2*^−/Y^ rats. Breathing abnormalities of the *Mecp2*^−/Y^ rats including irregular respiratory rhythm and high apnea rate resemble closely respiratory dysfunction in RTT patients and mouse models. Besides, LC neurons of *Mecp2*^−/Y^ rats show excessive firing activity, which has also been observed in the mouse models before. A half of the *Mecp2*^−/Y^ rats died in 2 months. These RTT-like symptoms observed in *Mecp2*^−/Y^ rats thus are comparable to those seen in *Mecp2*^−/Y^ mice [23, 28, 31]. Consistently, a recent study has demonstrated that the *Mecp2*^−/+^ rats had similarly impaired auditory responses as *Mecp2* heterozygous female mice [35, 36], which is seen in RTT patients as well [37]. Therefore, *Mecp2*^−/Y^ rats recapitulated most of the RTT-like symptoms, making them a quality RTT model.

### Onset time of RTT-like symptoms in *Mecp2*^−/Y^ rats

RTT is characterized by relatively normal development at the earlier stage of life. The asymptomatic time window is usually from birth to 6 months in humans with RTT [38]. In mouse models, the onset time and the disease progression vary depending on the nature of *Mecp2* variants and genetic background of mice. Most studies agree that *Mecp2*^−/Y^ mice have no overt RTT symptoms within the first 3 weeks of life. After that, the null mice show rapid development and progression of RTT-like symptoms. Growth retardation can be observed in P4w [21, 39, 40]. Locomotor problems appear in P3w-P4w in mouse models [10, 41]. Social defects are observed as early as 4 weeks after birth in mice [42]. Breathing disturbances occur in null mice at P3w-P4w [21]. In *Mecp2*^−/Y^ rats, these RTT-like symptoms also take place as early as 3 weeks, and become obvious at P4w. Thus, the onset time of the RTT-like phenotypes in the *Mecp2*^−/Y^ rats is comparable to that in *Mecp2*^−/Y^ mice.

### Defects of neuronal activity in *Mecp2*^−/Y^ rats

LC neurons are the predominant source of noradrenergic modulation in the CNS [43, 44]. They project to various regions in the brain, including the forebrain, diencephalon, brainstem, cerebellum, and even the spinal cord [43, 44]. Defects of LC neurons lead to dysfunction of autonomic nervous system, consistent with the characteristic RTT-like symptoms. In *Mecp2*^−/Y^ mice, the LC neurons show electrophysiological defects as increased neuronal firing activity, which is likely to be a result of the impaired intrinsic membrane properties and the deficiency in GABA-ergic inhibition [24, 25, 45]. Similar hyperexcitability of LC neurons is found in *Mecp2*^−/Y^ rats, suggesting that the *Mecp2*^−/Y^ rats may develop similar neuronal defects as *Mecp2*^−/Y^ mice. In addition to LC neurons, neuronal hyperexcitability is seen in neurons in the hippocampus, neocortex and other brainstem areas in *Mecp2*^−/Y^ mice, which contributes to the defects in motor function, cognition, sleep, and other autistic features [17, 46, 47]. These, though still unclear, may occur in other neurons of the *Mecp2*^−/Y^ rats.

### Symptomatic difference between *Mecp2*^−/Y^ rats and mice

Although the *Mecp2*^−/Y^ rat model has many similarities to the *Mecp2*^−/Y^ mouse models, our studies suggest that the former is not a complete replica of the latter. Indeed, some RTT-like phenotypes are quite different between rat and mouse models. All *Mecp2*^−/Y^ rats displayed malocclusion that appears more severe than null mice. The incisor teeth overly grow which may be due to the reduction of the central command of chewing activity, sick jaw muscles, and/or abnormal propriosensation in the transgenic rats. Besides, the *Mecp2*^−/Y^ rats had a proportional reduction in body size compared to their WT littermates, which seems more obvious than that seen in *Mecp2*^−/Y^ mice. Another difference found in over a half of *Mecp2*^−/Y^ rats is the penile prolapse. This symptom has been reported in transgenic mice with neurodegeneration [48] but not in *Mecp2*^−/Y^ mice. This often hinders the penis from retraction even with the lubrication treatment, which contributes to the humane endpoint of the rats. *Mecp2*^−/Y^ rats did not show the typical hindlimb grasping as *Mecp2*^−/Y^ mice did [49]. In the three-chamber test, old (P7w) *Mecp2*^−/Y^ rats barely switched between each chamber due to the poor locomotor activity, which has caused rejection of them from social behavioral tests. In contrast, mice do not show such age-dependent deterioration in locomotor activity as defects in social behaviors are reported at various ages from P4w to P12w in different mouse models [21, 50]. Furthermore, breathing abnormalities appear less severe in *Mecp2*^−/Y^ rats than in *Mecp2*^−/Y^ mice. This may be related to the low breathing frequency of the rats. In general, these symptomatic differences between *Mecp2*^−/Y^ mice and *Mecp2*^−/Y^ rats are rather moderate and quantitative rather than qualitative.

### Advantages and the applications of the RTT rat model

The rat model has several potentials in RTT studies. The understanding of the link between RTT phenotypes and the neuronal cellular and network mechanisms relies on in-vivo approaches that are still difficult to be fulfilled in mice. The larger body size of rats may satisfy the requirement for the in-vivo studies. The larger body size may benefit certain tests that need repetitive and timely samplings, such as measurement of blood gases, electrolytes and hormonal levels in circulation blood and cerebrospinal fluid, monitoring of systemic physiological parameters and EEG recordings. The richness of the social and communication repertoire of rats may also benefit the behavior studies, such as cognitive processes, anxiety, social acoustic memory, cooperative behaviors and auditory responses [51]. Most importantly, studies on multiple animal models can benefit RTT research, especially when therapeutic agents are to be developed.

## Conclusions

Our studies indicate that the novel *Mecp2*^−/Y^ rats recapitulate numerous RTT-like symptoms as the *Mecp2*^−/Y^ mouse model do. The RTT rat model, as a valuable model in the studies of RTT, may provide a useful alternative in RTT studies when body size is considered to be a critical factor.

## Abbreviations

*aCSF* artificial cerebrospinal fluid, *LC* locus coeruleus, *MeCP2* methyl CpG binding protein 2, *RTT* Rett Syndrome, WT, wild-type

## References

[1] Hagberg B (2005). Rett syndrome: long-term clinical follow-up experiences over four decades. J Child Neurol.

[2] Neul JL (2012). The relationship of Rett syndrome and MECP2 disorders to autism. Dialogues Clin Neurosci.

[3] Weng SM, Bailey ME, Cobb SR (2011). Rett syndrome: from bed to bench. Pediatr Neonatol.

[4] Glaze DG (2005). Neurophysiology of Rett syndrome. J Child Neurol.

[5] Katz DM, Dutschmann M, Ramirez JM, Hilaire G (2009). Breathing disorders in Rett syndrome: progressive neurochemical dysfunction in the respiratory network after birth. Respir Physiol Neurobiol.

[6] Ogier M, Katz DM (2008). Breathing dysfunction in Rett syndrome: understanding epigenetic regulation of the respiratory network. Respir Physiol Neurobiol.

[7] Rohdin M, Fernell E, Eriksson M, Albage M, Lagercrantz H, Katz-Salamon M (2007). Disturbances in cardiorespiratory function during day and night in Rett syndrome. Pediatr Neurol.

[8] Liyanage VR, Rastegar M (2014). Rett syndrome and MeCP2. Neuromolecular Med.

[9] Pelka GJ, Watson CM, Radziewic T, Hayward M, Lahooti H, Christodoulou J, Tam PP (2006). Mecp2 deficiency is associated with learning and cognitive deficits and altered gene activity in the hippocampal region of mice. Brain.

[10] Guy J, Hendrich B, Holmes M, Martin JE, Bird A (2001). A mouse Mecp2-null mutation causes neurological symptoms that mimic Rett syndrome. Nat Genet.

[11] Chen RZ, Akbarian S, Tudor M, Jaenisch R (2001). Deficiency of methyl-CpG binding protein-2 in CNS neurons results in a Rett-like phenotype in mice. Nat Genet.

[12] Goffin D, Allen M, Zhang L, Amorim M, Wang IT, Reyes AR, Mercado-Berton A, Ong C, Cohen S, Hu L (2012). Rett syndrome mutation MeCP2 T158A disrupts DNA binding, protein stability and ERP responses. Nat Neurosci.

[13] Schaevitz LR, Gomez NB, Zhen DP, Berger-Sweeney JE (2013). MeCP2 R168X male and female mutant mice exhibit Rett-like behavioral deficits. Genes Brain Behav.

[14] Yasui DH, Gonzales ML, Aflatooni JO, Crary FK, Hu DJ, Gavino BJ, Golub MS, Vincent JB, Carolyn Schanen N, Olson CO (2014). Mice with an isoform-ablating Mecp2 exon 1 mutation recapitulate the neurologic deficits of Rett syndrome. Hum Mol Genet.

[15] Samaco RC, Neul JL (2011). Complexities of Rett syndrome and MeCP2. J Neurosci.

[16] Chao HT, Chen H, Samaco RC, Xue M, Chahrour M, Yoo J, Neul JL, Gong S, Lu HC, Heintz N (2010). Dysfunction in GABA signalling mediates autism-like stereotypies and Rett syndrome phenotypes. Nature.

[17] Kron M, Howell CJ, Adams IT, Ransbottom M, Christian D, Ogier M, Katz DM (2012). Brain activity mapping in Mecp2 mutant mice reveals functional deficits in forebrain circuits, including key nodes in the default mode network, that are reversed with ketamine treatment. J Neurosci.

[18] Joynt KE, Orav EJ, Jha AK (2013). Physician volume, specialty, and outcomes of care for patients with heart failure. Circ Heart Fail.

[19] Zhong W, Cui N, Jin X, Oginsky MF, Wu Y, Zhang S, Bondy B, Johnson CM, Jiang C (2015). Methyl CpG binding protein 2 gene disruption augments tonic currents of gamma-aminobutyric acid receptors in locus coeruleus neurons: IMPACT ON NEURONAL EXCITABILITY AND BREATHING. J Biol Chem.

[20] Voituron N, Zanella S, Menuet C, Dutschmann M, Hilaire G (2009). Early breathing defects after moderate hypoxia or hypercapnia in a mouse model of Rett syndrome. Respir Physiol Neurobiol.

[21] Viemari JC, Roux JC, Tryba AK, Saywell V, Burnet H, Pena F, Zanella S, Bevengut M, Barthelemy-Requin M, Herzing LB (2005). Mecp2 deficiency disrupts norepinephrine and respiratory systems in mice. J Neurosci.

[22] Lioy DT, Wu WW, Bissonnette JM (2011). Autonomic dysfunction with mutations in the gene that encodes methyl-CpG-binding protein 2: insights into Rett syndrome. Auton Neurosci.

[23] Zhang X, Su J, Cui N, Gai H, Wu Z, Jiang C (2011). The disruption of central CO2 chemosensitivity in a mouse model of Rett syndrome. Am J Physiol Cell Physiol.

[24] Zhang X, Cui N, Wu Z, Su J, Tadepalli JS, Sekizar S, Jiang C (2010). Intrinsic membrane properties of locus coeruleus neurons in Mecp2-null mice. Am J Physiol Cell Physiol.

[25] Jin X, Cui N, Zhong W, Jin XT, Jiang C (2013). GABAergic synaptic inputs of locus coeruleus neurons in wild-type and Mecp2-null mice. Am J Physiol Cell Physiol.

[26] Weaving LS, Williamson SL, Bennetts B, Davis M, Ellaway CJ, Leonard H, Thong MK, Delatycki M, Thompson EM, Laing N, Christodoulou J (2003). Effects of MECP2 mutation type, location and X-inactivation in modulating Rett syndrome phenotype. Am J Med Genet A.

[27] Young JI, Zoghbi HY (2004). X-chromosome inactivation patterns are unbalanced and affect the phenotypic outcome in a mouse model of rett syndrome. Am J Hum Genet.

[28] Silverman JL, Yang M, Lord C, Crawley JN (2010). Behavioural phenotyping assays for mouse models of autism. Nat Rev Neurosci.

[29] Kaidanovich-Beilin O, Lipina T, Vukobradovic I, Roder J, Woodgett JR. Assessment of social interaction behaviors. J Vis Exp. 2011; 48. pii: 2473. 10.3791/2473 PMC3197404 21403628

[30] Moy SS, Nadler JJ, Perez A, Barbaro RP, Johns JM, Magnuson TR, Piven J, Crawley JN (2004). Sociability and preference for social novelty in five inbred strains: an approach to assess autistic-like behavior in mice. Genes Brain Behav.

[31] Abdala AP, Dutschmann M, Bissonnette JM, Paton JF (2010). Correction of respiratory disorders in a mouse model of Rett syndrome. Proc Natl Acad Sci U S A.

[32] Taneja P, Ogier M, Brooks-Harris G, Schmid DA, Katz DM, Nelson SB (2009). Pathophysiology of locus ceruleus neurons in a mouse model of Rett syndrome. J Neurosci.

[33] Schanen NC, Kurczynski TW, Brunelle D, Woodcock MM, Dure LS, Percy AK (1998). Neonatal encephalopathy in two boys in families with recurrent Rett syndrome. J Child Neurol.

[34] Wan M, Lee SS, Zhang X, Houwink-Manville I, Song HR, Amir RE, Budden S, Naidu S, Pereira JL, Lo IF (1999). Rett syndrome and beyond: recurrent spontaneous and familial MECP2 mutations at CpG hotspots. Am J Hum Genet.

[35] Engineer CT, Rahebi KC, Borland MS, Buell EP, Centanni TM, Fink MK, Im KW, Wilson LG, Kilgard MP (2015). Degraded neural and behavioral processing of speech sounds in a rat model of Rett syndrome. Neurobiol Dis.

[36] Liao W, Gandal MJ, Ehrlichman RS, Siegel SJ, Carlson GC (2012). MeCP2+/- mouse model of RTT reproduces auditory phenotypes associated with Rett syndrome and replicate select EEG endophenotypes of autism spectrum disorder. Neurobiol Dis.

[37] Bader GG, Witt-Engerstrom I, Hagberg B (1989). Neurophysiological findings in the Rett syndrome, II: visual and auditory brainstem, middle and late evoked responses. Brain Dev.

[38] Hagberg B, Aicardi J, Dias K, Ramos O (1983). A progressive syndrome of autism, dementia, ataxia, and loss of purposeful hand use in girls: Rett’s syndrome: report of 35 cases. Ann Neurol.

[39] Calfa G, Percy AK, Pozzo-Miller L (2011). Experimental models of Rett syndrome based on Mecp2 dysfunction. Exp Biol Med (Maywood).

[40] Stettner GM, Huppke P, Gartner J, Richter DW, Dutschmann M (2008). Disturbances of breathing in Rett syndrome: results from patients and animal models. Adv Exp Med Biol.

[41] Santos M, Silva-Fernandes A, Oliveira P, Sousa N, Maciel P (2007). Evidence for abnormal early development in a mouse model of Rett syndrome. Genes Brain Behav.

[42] Castro J, Garcia RI, Kwok S, Banerjee A, Petravicz J, Woodson J, Mellios N, Tropea D, Sur M (2014). Functional recovery with recombinant human IGF1 treatment in a mouse model of Rett syndrome. Proc Natl Acad Sci U S A.

[43] Samuels ER, Szabadi E (2008). Functional neuroanatomy of the noradrenergic locus coeruleus: its roles in the regulation of arousal and autonomic function part I: principles of functional organisation. Curr Neuropharmacol.

[44] Samuels ER, Szabadi E (2008). Functional neuroanatomy of the noradrenergic locus coeruleus: its roles in the regulation of arousal and autonomic function part II: physiological and pharmacological manipulations and pathological alterations of locus coeruleus activity in humans. Curr Neuropharmacol.

[45] Jin X, Zhong W, Jiang C (2013). Time-dependent modulation of GABA(A)-ergic synaptic transmission by allopregnanolone in locus coeruleus neurons of Mecp2-null mice. Am J Physiol Cell Physiol.

[46] Zhang W, Peterson M, Beyer B, Frankel WN, Zhang ZW (2014). Loss of MeCP2 from forebrain excitatory neurons leads to cortical hyperexcitation and seizures. J Neurosci.

[47] Zhang L, He J, Jugloff DG, Eubanks JH (2008). The MeCP2-null mouse hippocampus displays altered basal inhibitory rhythms and is prone to hyperexcitability. Hippocampus.

[48] Pacheco CD, Elrick MJ, Lieberman AP (2009). Tau deletion exacerbates the phenotype of Niemann-Pick type C mice and implicates autophagy in pathogenesis. Hum Mol Genet.

[49] Guy J, Gan J, Selfridge J, Cobb S, Bird A (2007). Reversal of neurological defects in a mouse model of Rett syndrome. Science.

[50] Kerr B, Alvarez-Saavedra M, Saez MA, Saona A, Young JI (2008). Defective body-weight regulation, motor control and abnormal social interactions in Mecp2 hypomorphic mice. Hum Mol Genet.

[51] Wohr M, Scattoni ML (2013). Behavioural methods used in rodent models of autism spectrum disorders: current standards and new developments. Behav Brain Res.

